# 

**Published:** 1883-04

**Authors:** 


### BOOKS AND PAMPHLETS RECEIVED.

### BOOKS.

Quain's Anatomy. Ninth Edition, 2 Vols- N. Y., Wm. Wood & Co. Chicago, W. T. Keener.

Manual of Histology. Satterthwaite. Second Edition. N. Y., Wm. Wood & Co. Chicago, W. T. Keener.

Diseases of the Prostate. Thompson. Phila., P. Blakiston, Son & Co. Chicago, W. T. Keener.

Vision, its Optical Defects. Fenner. Second Edition. Phila., P. Blakiston, Son & Co. Chicago, W. T. Keener.

Manual of Gynæcology. Hart and Barbour. N. Y., Wm. Wood & Co., Med. Lib. Chicago, W. T. Keener.

The Untoward Effects of Drugs. Lewin. Detroit, G. S. Davis.

Experimental Pharmacology. L. Hermann Zurich. Lea's Son & Co. Chicago, Jansen, McClurg & Co.

Nerve Prostration and Hysteria. W. S. Playfair, London. Phila., Lea's Son & Co. Chicago, Jansen, McClurg & Co.

Physical Exploration of the Lungs. Flint. Phila., H. C. Lea's Son & Co. Jansen, McClurg & Co.

Chronic Bronchitis. Bartholow. W. T. Keener.

The Uterus, Ovaries and Fallopian Tubes. Courty. N. Y., Blakiston, Son & Co. W. T. Keener.

PAMPHLETS.

Modified Listerism in Ovariotomy. Edw. W. Jenks.

A Large Fibro-Cyst of the Uterus and Ovarian Cystoma, with Pregnancy. Operation. Recovery. Walter Coles.

Remarks on 177 Operations for Entropium and Trichiasis. F. C. Hotz.

Transactions State Medical Society of Arkansas. 1882.

Contributions from the Chemical Laboratory of the University of Michigan. Prescott & Vaughan.

The Treatment of Acute Eczema. Geo. H. Rohe.

Bacteria and their Presence in Syphilitic Secretions. R. H. Morrison.

Percentage of College-bred Men in the Medical Profession. Chas. McIntire.

The Clinical Diagnosis of Bright's Disease. C. W. Purdy.

Annual Report Marine Hospital Service. 1882.

Traumatic Tetanus. Recovery under Quinine and Alcohol. Sphæro-Bacteria Present in the Blood. D. R. Brower.

Micro-Organisms in the Blood of Tetanus. Lester Curtis.

Preliminary Report on the Yellow Fever Epidemic of 1882 in the State of Texas. Treasury Department.

On the Question of Hypertrophy of the Osseous Structure of the Turbinated Bones. D. Bryson Delavan.

Bromide of Ethyl. Julian J. Chisolm.

Announcement Toledo Medical College. 1883.

What Shall We Do for the Drunkard? Orpheus Everts.

Annual Address before the American Academy of Medicine, Philadelphia. Triall Green.

Uterine Massage. A. Reeves Jackson.

Tracheotomy in Illinois. H. Z. Gill.

Treatment of Puerperal Septicæmia by Intra-Uterine Injections. Edw. Jenks. 1880.

Best Methods of Treating Operative Wounds. Henry O. Marcy.

Deflection of the Septum Narium. E. Fletcher Ingals.

Obstructions in the Larynx and Trachea. E. Fletcher Ingals.

Announcement Cooper Medical College, San Francisco. Session 1883.

Announcement of Spring Course, College of Physicians and Surgeons. 1883.

Management of Chronic Inebriates. Albert N. Blodgettt.

Transactions of the American Dermatological Association. 1882. Arthur Van Harlingen, Secretary.

A Simple Suction Drainage Tube for Suppurating Pleural Cavities. Edmund Andrews.

On Prehistoric Trephining and Historic Amulets. Robert Fletcher.

Contribution to Surgical Gynæcology. Edw. W. Jenks.

Present Status of Antiseptic Surgery. R. Park.

Stricture of the Rectum Treated by Electrolysis. R. Newman.

Medical Communications of the Massachusetts Medical Society. Vol. XIII, No. 1. 1882.

Analysis of Eight Thousand Cases of Skin Disease. L. D. Bulkley.

Naso-Antral Catarrh and Its Treatment. W. N. Daly.

Transactions Medical Society of West Virginia. Fourteenth and Fifteenth Annual Sessions. 1881 and 1882.

Address in Surgery, Excisions of Portions of the Alimentary Canal Covered by Peritoneum. W. A. Byrd.

Life of John M. Briggs. W. K. Bowling.

Early Diagnosis of Chronic Bright's Disease. T. A. McBride.

Placental Delivery in Mammals. Henry O. Marcy.

Addresses on the Dedication of Cooper Medical College Building. By Levi C. Lane and Edw. R. Taylor.

Fifty-seventh Annual Report of the Massachusetts Charitable Eye and Ear Infirmary. 1882.

Investigation into the Parasites in the Pork Supply of Montreal. Osler and Clement.

Value of Graduated Pressure in Diseases of the Vagina, Uterus, Ovaries and Other Appendages. Nathau Bozeman.

Laryngeal Hæmorrhage. J. Bettman.

Amnesic Aphasia. E. Wyllys Andrews.

Second Annual Report of the Astronomer of Yale College Observatory. 1882.

The Yellow Oxide of Mercury. W. W. Seeley.

Summary of Monthly Medical Reports Southern Illinois Penitentiary. H. Z. Gill.

The Differential Diagnosis of the Cause of Sudden Unconsciousness. R. O. Beard.

Glycosuria. O. C. DeWolf.

Some Points on the Administration of Anæsthetics. Geo. H. Rohe.

First Biennial Report of the Michigan Free Eye and Ear Infirmary. 1882.

The Growth of Children. Geo. W. Peckham.

Transactions of the Michigan State Medical Society. 1882.

The City of Mobile as a Winter Resort. W. H. Anderson.

The Antiseptic Treatment of Wounds. W. T. Briggs.

Restraint and Seclusion in American Institutions for the Insane. H. M. Bannister and H. N. Moyer.

Elephantiasis Arabum in the Samoan Islands. A. C. Heffinger.

Meteorology and Rainfall. J. F. Bateman.

Remarks on Climate in Relation to Organic Nature. Surgeon-General C. A. Gordon, M.D., C.B.

The Malignity of Syphilis. L. D. Bulkley.

---

### THE TREATMENT OF HYDROPS WITH BLATTA ORIENTALIS.

In the Medical Society of St. Petersburg, Dr. Bogomslow reported his experience with this drug in cases of hydrops. The latter was produced by disease of the heart in fifteen cases; by diseases of the kidney fifty-two times, and in three persons by degeneration of the liver. In twenty-nine cases the remedy was given as a powder, and as a tincture in the others. Profuse sweating was produced in nineteen cases, while in sixty-one there was increased urination, and in thirteen diarrhœa was provoked. —*St. Petersburg Med. Wochenschrift.*

CLINICS.

MONDAY.

Eye and Ear Infirmary—1.15 to 2.15 p. m., Otological, by Dr. Schaefer; 2.15 to 3.30 p. m., Ophthalmological, Dr. Holmes.
Mercy Hospital—2 p. m., Medical, Profs. Hollister and Quine.
Rush Medical College—2 p. m., Medical by Prof. Bridge; 3 p. m., Dermatological and Venereal, by Prof. Hyde.
Woman's Medical College—2 p. m., Dermatological and Venereal, by Prof. Maynard.

TUESDAY.

Cook Co. Hospital—2 to 4 p. m., Medical and Surgical Clinics.
Mercy Hospital—2 p. m., Surgical Clinic, by Prof. Andrews.
Woman's Medical College—10 a. m., Prof. Ingals.

WEDNESDAY.

Chicago Medical College—2 p. m., Eye and Ear, by Prof. Jones.
Rush Medical College—3 p. m., Ophthalmological and Otological, by Prof. Holmes: 3 to 4 p. m., Diseases of the Chest, by Prof. Ross.
Woman's Medical College—2 p. m., Eye and Ear, by Dr. W. T. Montgomery; 3 p. m., Diseases of Children, by Prof. Chas. W. Earle.
Eye and Ear Infirmary—2.30 p. m.. Dr. E. J. Gardiner.

THURSDAY.

Chicago Medical College—2 p. m.. Gynæcological, Prof. Dudley.
Rush Medical College—2 p. m., Diseases of Children, by Prof. Knox; 3 p. m., Diseases of the Nervous System, by Prof. Lyman.
Eye and Ear Infirmary—1.15 to 2.25 p. m., Otological. by Dr. Schaefer; 2.15 to 3.30, p. m., Ophthalmological, Dr. Holmes.
Woman's Medical College—3 p. m., Surgical, by Prof. Owens.
College of Physicians and Surgeons—2 p. m., Medical, by Prof. S. A. McWilliams; 3 p. m., Surgical, by Prof. R. L. Rea.

FRIDAY.

Cook County Hospital—2 to 4 p. m., Medical and Surgical Clinics.
Mercy Hospital—2 p. m., Medical, by Prof. Davis.

SATURDAY.

Rush Medical College—2 p. m., Surgical, by Prof. Gunn.
Mercy Hospital—2 p. m., Surgical Clinic, by Prof. Andrews.
Chicago Medical College—3 p. m., Neurological, Prof. Jewell.
Woman's Medical College—2 p. m., Gynæcological, by Prof. T. D. Fitch.
College of Physicians and Surgeons—2 p. m., Diseases of the Chest, by Prof. S. A. McWilliams; 3 p. m., Gynæcological, by Prof. A. Reeves Jackson.

Daily Clinics, from 2 to 4 p. m., at the Central Free Dispensary, at the South Side Dispensary and at the West Side Dispensary.

